# Transcriptomic Signature of Right Ventricular Failure in Experimental Pulmonary Arterial Hypertension: Deep Sequencing Demonstrates Mitochondrial, Fibrotic, Inflammatory and Angiogenic Abnormalities

**DOI:** 10.3390/ijms19092730

**Published:** 2018-09-12

**Authors:** Francois Potus, Charles Colin Thomas Hindmarch, Kimberly J. Dunham-Snary, Jeff Stafford, Stephen L. Archer

**Affiliations:** 1 Department of Medicine, Queen’s University, Kingston, ON K7L3N6, Canada; FP17@queensu.ca (F.P.); kimberly.dunhamsnary@queensu.ca (K.J.D.-S.); 2 Queen’s Cardiopulmonary Unit (QCPU), Translational Institute of Medicine (TIME), Department of Medicine, Queen’s University, Kingston, ON K7L3N6, Canada; c.hindmarch@queensu.ca; 3 Centre for Advanced Computing, Queen’s University, Kingston, ON K7L3N6, Canada; jeff.stafford@queensu.ca

**Keywords:** transcriptomics, mitochondria, metabolism, *TFAM*, *CYP2E1*, *GALNT13*, *ANXA1*, *periostin*, *LTBP2*, *THBS4*

## Abstract

Right ventricular failure (RVF) remains the leading cause of death in pulmonary arterial hypertension (PAH). We investigated the transcriptomic signature of RVF in hemodynamically well-phenotyped monocrotaline (MCT)-treated, male, Sprague-Dawley rats with severe PAH and decompensated RVF (increased right ventricular (RV) end diastolic volume (EDV), decreased cardiac output (CO), tricuspid annular plane systolic excursion (TAPSE) and ventricular-arterial decoupling). RNA sequencing revealed 2547 differentially regulated transcripts in MCT-RVF RVs. Multiple enriched gene ontology (GO) terms converged on mitochondria/metabolism, fibrosis, inflammation, and angiogenesis. The mitochondrial transcriptomic pathway is the most affected in RVF, with 413 dysregulated genes. Downregulated genes included *TFAM* (−0.45-fold), suggesting impaired mitochondrial biogenesis, *CYP2E1* (−3.8-fold), a monooxygenase which when downregulated increases oxidative stress, dehydrogenase/reductase 7C (*DHRS7C*) (−2.8-fold), consistent with excessive autonomic activation, and polypeptide *N*-acetyl-galactose-aminyl-transferase 13 (*GALNT13*), a known pulmonary hypertension (PH) biomarker (−2.7-fold). The most up-regulated gene encodes Periostin (*POSTN*; 4.5-fold), a matricellular protein relevant to fibrosis. Other dysregulated genes relevant to fibrosis include latent-transforming growth factor beta-binding protein 2 (*LTBP2*), thrombospondin4 (*THBS4*). We also identified one dysregulated gene relevant to all disordered transcriptomic pathways, *ANNEXIN A1*. This anti-inflammatory, phospholipid-binding mediator, is a putative target for therapy in RVF-PAH. Comparison of expression profiles in the MCT-RV with published microarray data from the RV of pulmonary artery-banded mice and humans with bone morphogenetic protein receptor type 2 (*BMPR2*)-mutations PAH reveals substantial conservation of gene dysregulation, which may facilitate clinical translation of preclinical therapeutic and biomarkers studies. Transcriptomics reveals the molecular fingerprint of RVF to be heavily characterized by mitochondrial dysfunction, fibrosis and inflammation.

## 1. Introduction

Right ventricular (RV) function is the most important predictor of morbidity and mortality in pulmonary arterial hypertension (PAH) [1]. Current therapeutics target the pulmonary vasculature and while they provide important functional improvement, PAH survival remains poor (~50% at 5-years) [2,3,4]. A better understanding of RVF and identification of new therapeutic targets is crucial to improving patient survival.

RVF results from the dysfunction of multiple overlapping systems, including mitochondria, metabolism, fibrosis, inflammation, and angiogenesis [5,6]. Fibrosis, characterized by collagen accumulation and excessive extracellular remodelling is closely linked with increased RV stiffness and subsequent deterioration of RV function [7]. There is a clear correlation between RV fibrosis and reduced RV function [8,9,10]. Conversely, reductions in fibrosis correlate with improvements in RV function [11,12].

Inflammation also contributes to RVF. Increased plasma levels of C-reactive protein (CRP) and the inflammatory cytokine interleukin-6 (IL-6) are associated with reduced RV function in human PAH [13,14]. Scleroderma-associated PAH (SSc-PAH) is a condition in which autoimmunity is a hallmark. Affected patients display RV dysfunction that is disproportionate to PA pressure and have a worse prognosis than patients with idiopathic PAH [15].

Angiogenesis, a process closely related to inflammation and fibrosis [16,17], is disrupted in PAH, resulting in reduced capillary density in both the pulmonary vasculature and RV. Capillary rarefaction in the RV is associated with dysregulation of VEGF, Angiopoietin and SPRED-1 [6,18,19]. Conversely, increased angiogenesis and RV capillary density by epigenetic downregulation of SPRED-1 in a preclinical model of PAH is associated with improved RV function and decreased fibrosis [19]. Moreover, virus-mediated delivery of angiogenic factors is protective in animal models of PAH [20,21].

Our group has previously reported the contribution of mitochondrial/metabolic dysregulation to the aetiology of RVF in PAH. Interestingly, there are parallel mitochondrial/metabolic abnormalities in the pulmonary vasculature and heart in PAH, making this a particularly appealing therapeutic target. RVF is associated with a metabolic shift away from oxidative fatty acid or glucose metabolism toward a pattern of metabolism characterized by increased uncoupled, aerobic glycolysis. This Warburg metabolism is multifactorial caused in part by upregulation of pyruvate dehydrogenase kinase (PDK) and inhibition of phosphodiesterase of pyruvate dehydrogenase (PDH). There is also increased glutaminolysis (reflecting ischemia-induced cMyc activation) in RVF and abnormalities in mitochondrial complexes II, III and IV (CXI; II; IV) [22,23,24]. Decreased mitochondrial density and organellar structural changes have also been reported in RV of PAH animal models [25,26]. It is not only the RV myocyte and coronary arteries that are adversely affected in the PAH RV; we have demonstrated that RV fibroblasts from monocrotaline (MCT) rats display increased mitochondrial fission, which is associated with increased fibroblast proliferation and collagen deposition [27].

Here, we provide a comprehensive hemodynamic of RVF and demonstrate the corresponding transcriptomic signature of RVF in the monocrotaline-induced (MCT)-PAH model [28]. Using this unbiased strategy, we show marked dysregulation of four pathways: mitochondria/metabolism, fibrosis, inflammation and angiogenesis.

## 2. Results

### 2.1. Characterization of RVF in MCT-PAH

**Figure 1:**
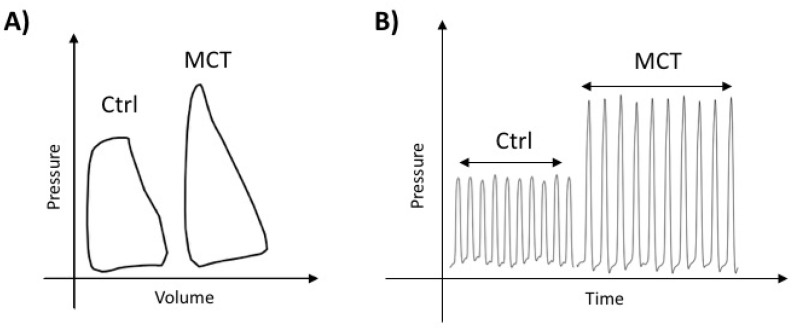
(**A**) Representative pressure volume loop and (**B**) pressure trace in monocrotaline (MCT) and control (Ctrl) animals.

RV function was quantified using non-invasive echocardiography prior to sacrifice and a terminal right heart catheterization (RHC), both performed in anesthetised, closed-chest rats (Figure 1A,B and Table 1). Representative PV-loop and pressure measurements from Ctrl and MCT rats are shown in Figure 1A,B. Compared to control, MCT displayed significant pulmonary hypertension, characterized by increased mean pulmonary arterial pressure (mPAP), right ventricular systolic pressure (RVSP), total pulmonary resistance (TPR), arterial elastance (Ea) and shortened pulmonary artery acceleration time (PAAT). MCT rats displayed right ventricular hypertrophy (RVH), as assessed by the Fulton index and right ventricle free wall (RVFW) thickness, and increased end diastolic volume (EDV). MCT rats had a significant decrease in cardiac output (CO). MCT animals displayed systolic dysfunction characterized by decreased tricuspid annular plane systolic excursion (TAPSE), ejection fraction (EF), and increased peak rate pressure decline (dP/dt_max_) and stroke work (SW) as well as diastolic dysfunction suggested by increased dP/dt_max_ and Tau Mirnsky. Independent of pre- and afterload, RVs from MCT rats had decreased contractility (increased preload recruited stroke work (PRSW) and pressure volume area-EDV relationship (PVA/EDV)) and impaired cardiovascular efficiency (decreased ventricular-arterial coupling, ventricular arterial coupling (Emax/Ea)), indicating that MCT RV dysfunction is not solely caused by increased afterload (Table 1).

### 2.2. RVF Is Associated with Increased Fibrosis, Inflammation Decreased Angiogenesis and Mitochondrial/Metabolic Dysfunction

**Figure 2:**
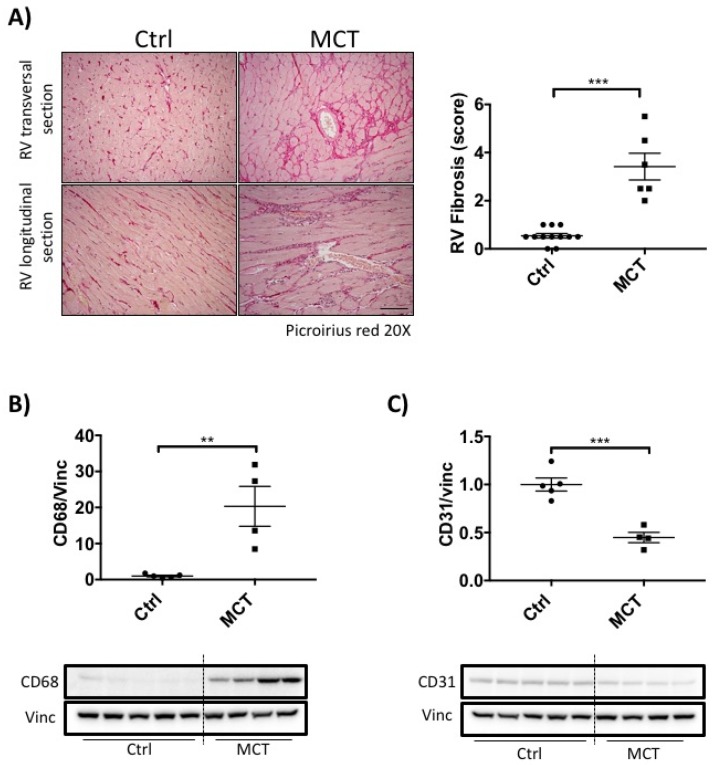
Right heart failure is associated with increased fibrosis, inflammation and angiogenic defect. (**A**) Fibrosis assessed by picrosirius red staining of RV from MCT (*n* = 8) vs. Ctrl (*n* = 15) rats. Scale bar in black represent 100 µm; (**B**) Inflammation assessed via CD68 immunoblot (*n* ≥ 4 per group); (**C**) Capillary density assessed by CD31 immunoblot (*n* ≥ 4 per group). Unpaired *t*-test; data expressed as mean ± SEM. ** *p* < 0.01; *** *p* < 0.001.

**Figure 3:**
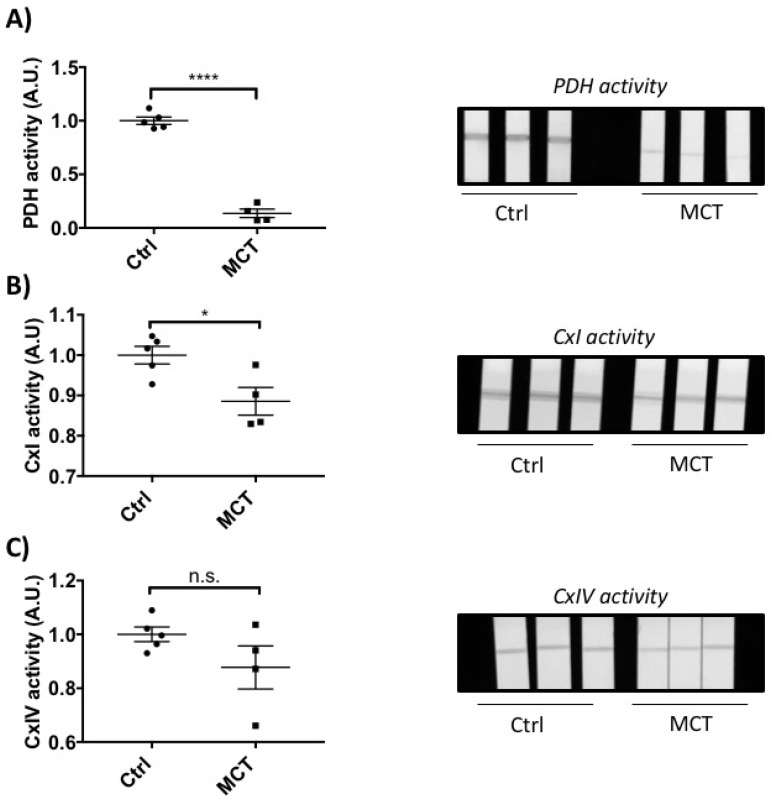
Right heart failure is associated with metabolic and mitochondrial dysfunction. (**A**) pyruvate dehydrogenase (PDH) activity; (**B**) mitochondrial complex I (CxI); and (**C**) mitochondrial complex IV (CxIV) activity, all measured via dipstick assay (*n* ≥ 4 per group). Unpaired *t*-test; data expressed as mean ± SEM. n.s.—not significant, * *p* < 0.05; **** *p* < 0.0001.

Confirming previous reports [5], MCT-RVF was associated with RV fibrosis, as assessed by increased collagen deposition (Figure 2A). Inflammation was also increased in MCT RVs, as measured by an increase in CD68 (monocyte cell marker, Figure 2B). MCT also resulted in RV capillary rarefaction, evident from the global diminution of CD31, an endothelial cell marker (Figure 2C). There was a significant functional mitochondrial defect in MCT RVs, indicated by decreased activity of PDH, Complex I and Complex IV (Figure 3A–C).

### 2.3. RNA Sequencing

RNA sequencing revealed 2546 transcripts that were significantly and differentially regulated in the RV as a consequence of MCT treatment (Table 2, Table S1). To validate this sequencing-derived dataset, we used quantitative polymerase chain reaction (PCR) and successfully validated the 6 most significantly and differentially regulated genes in the arrays, using cDNA extracted from an independent group of animals (Table 3).

### 2.4. Functional Annotation of the MCT RV Transcriptome

In order to understand the global function of our data, we performed a Functional Annotation analysis using the Database for Annotation, Visualization and Integrated Discovery (DAVID) [29]. This revealed multiple enriched terms, many of which converged on functions that describe mitochondria/metabolism, fibrosis and inflammation (Table 2, Table S2). The most significant altered gene ontology (GO) terms related to mitochondria (GO: 0005739; *p* = 3.5 × 10^−53^). There were 413 dysregulated mitochondrial-relevant genes in the MCT RV. We validated 15 of the expression changes with qPCR in an independent group of animals (Table 4, Table S1). We then filtered this list and identified a broad list of genes annotated under: Mitochondria/Metabolism, Fibrosis and Inflammation (Table S3). “Angiogenesis” did not appear via DAVID analysis, so a gene list associated with the Gene Ontology term Angiogenesis (GO: 0001525, Table S4) was imported and our MCT regulated gene list sorted accordingly (Table S1).

### 2.5. Comparison with Mouse and Human RV Transcriptomic Data

**Figure 4:**
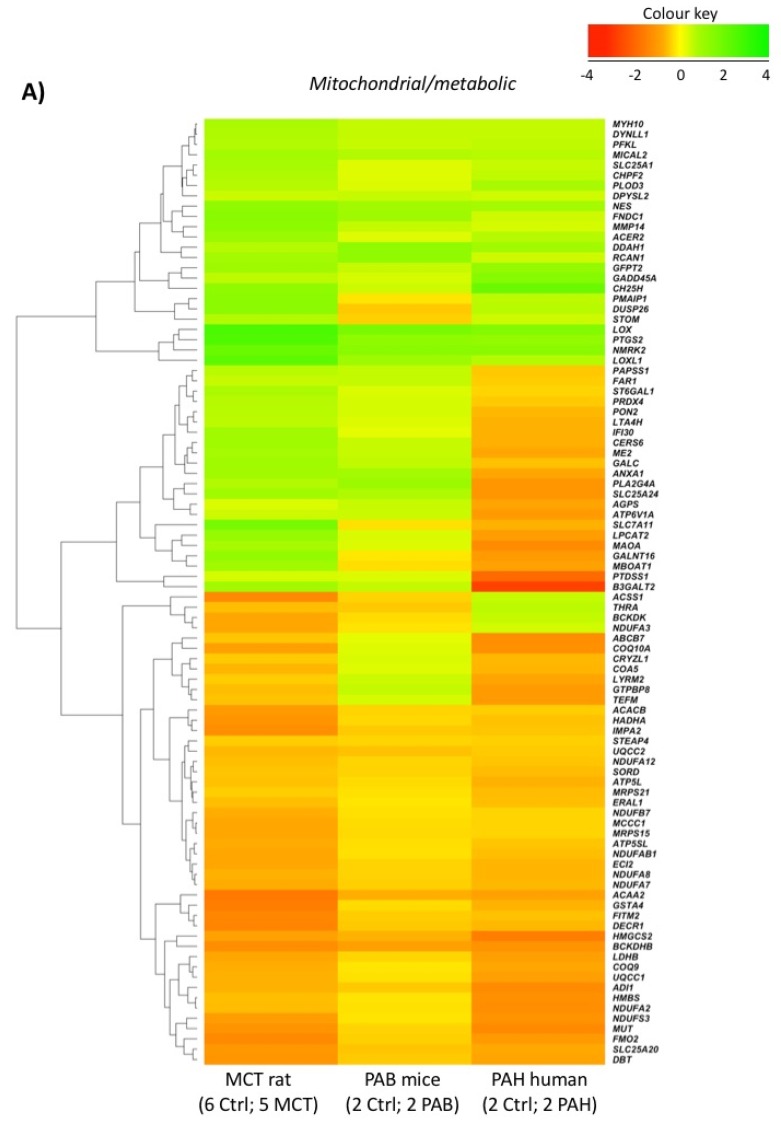

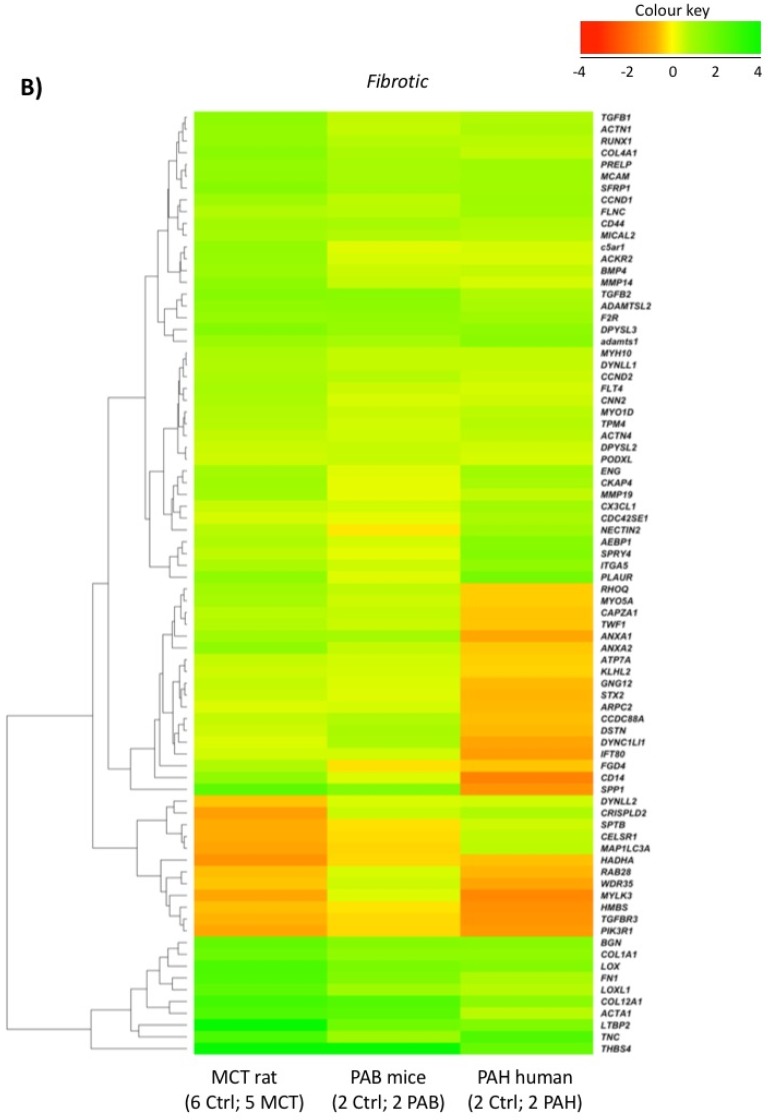

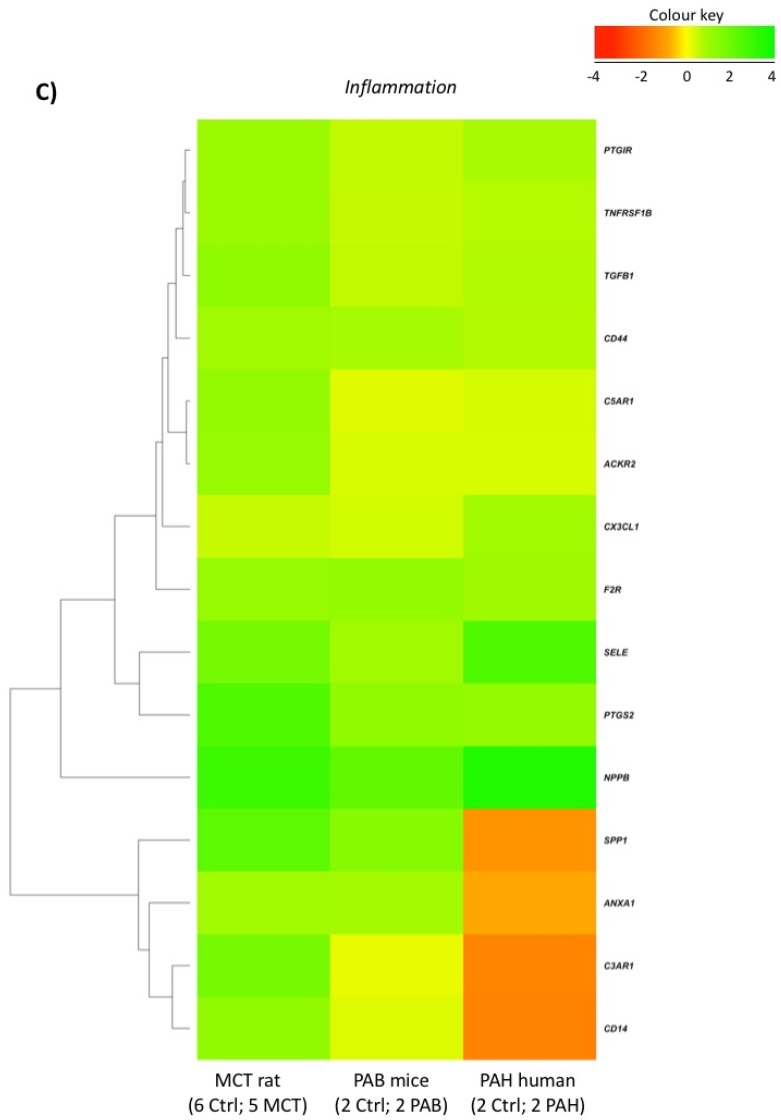

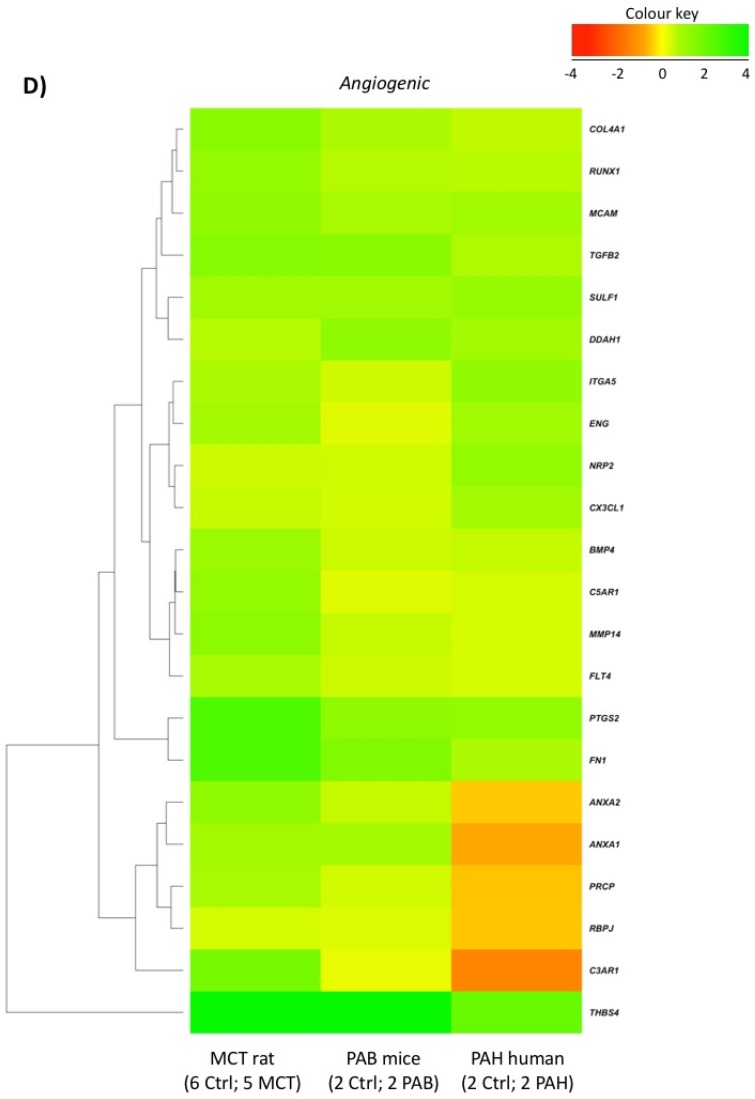
Gene expression changes in the MCT rat, PAB mouse and human *BMPR2*-PAH. Log2fold differentially-regulated genes in enriched lists: (**A**) Mitochondria/Metabolism, (**B**) Fibrosis, (**C**) Inflammation and (**D**) Angiogenesis, in the MCT rat RV, mouse pulmonary artery-banded RV (*n* = 2, GSE30428) [30], and human BMPR2-PAH (*n* = 2, GSE67492) [31]. Mouse and human microarrays analysed using NCBI GEOR2.

**Figure 5:**
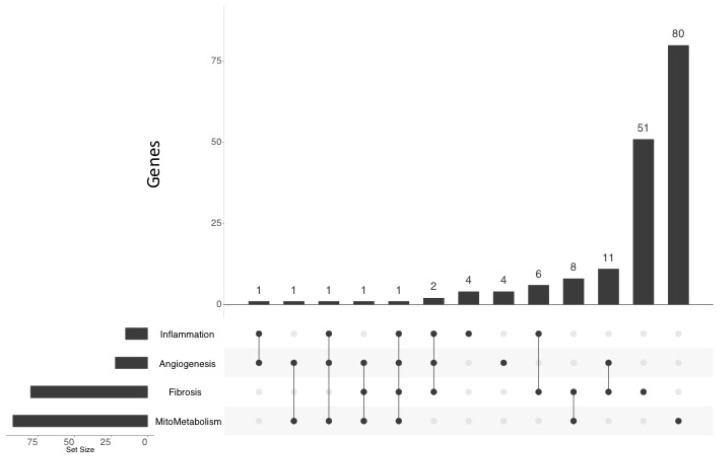
Intersection between inflammatory, angiogenic, fibrotic, and mitochondrial/metabolic gene expression. Data organized into a matrix and visualized using UpSetR, an R package that allows the resolution of multiple interactions to be made. Inflammation, Fibrosis, Mitochondria/Metabolism and Angiogenesis lists were compared to identify intersections in RV of the MCT rat according to function.

We mined published microarray data available from the National Centre for Biotechnology Information (NCBI) Gene-Expression-Omnibus (GEO). We obtained data from a mouse model of chronic RV outflow tract obstruction and RVH induced by pulmonary artery banding (PAB; 6-weeks, *n* = 2 and controls, *n* = 2; Accession: GSE30428; Table S5) [30] and from PAH patients with *BMPR2* mutations vs. controls (postmortem RV expression data, *n* = 2 per group; Accession: GSE67492; Table S6) [31]. We used GEO2R to identify differentially regulated genes in each of these experiments and compared these expression profiles to our rat MCT RV profile (Table S1). We identified genes as being common to rat and mouse (Table S7; 930) or rat and human (Table S8; 867) based on a loose *p*-value filter of *p* < 0.1. In total, 347 genes were commonly regulated between the rat MCT RV, mouse PAB RV and human *BMPR2* RV (Table S9). When these 347 genes were compared to the original annotated gene lists (Tables S3 and S4), 80 in “Fibroblast” (Table S10; Figure 4B), 92 were present in “Mitochondria/Metabolism” (Table S11; Figure 4A), 15 in “Inflammation” (Table S12; Figure 4C) and 22 in “Angiogenesis” (Table S13; Figure 4D). Several of these key genes were overlapping between pathways (Tables S3 and S4; Figure 5).

## 3. Discussion

The well-studied rat MCT model of PAH exhibits both RVF and pulmonary vascular remodeling. We performed several levels of phenotyping prior to the transcriptomic study. First, we created an extensive hemodynamic profile of this model, confirming that the rats were in the decompensated phased of RVF at the time the transcriptomics were measured. Careful phenotyping is, in our view, the inception point of any transcriptomic study. Evidence for RV decompensation include reduced TAPSE and increased RVEDP, and relatively low mPAP with high PVR. Moreover, through measurement of RV contractility, using PV-loop evaluation in closed-chest rats, we documented increased PRSW and PVA/EDV and a decreased Emax/Ea ratio in MCT-PAH, consistent with impaired RV-pulmonary arterial uncoupling (Table 1). These data are consistent with impaired RV myocardial contractility that is independent of both preload and afterload [32,33]. Since it is likely that the transcriptomic profile is different in RV compensation vs. decompensation, careful hemodynamic profiling is a strength of this study.

Second, we confirmed previous reports that RVF associated with PAH is characterized by increased fibrosis, inflammation and impaired angiogenesis [6]. We did this by documenting that in the MCT RV, there is increased picoserius red staining (collagen content), increased expression of the macrophage marker CD68 and decreased expression of the vascular endothelial marker CD31 (Figure 2A–C).

Third, we confirm that the RVs we studied manifested significant impairment of mitochondrial metabolism, demonstrated as a reduction in PDH activity (Figure 3A). Consistent with this, we report for the first time significant reduced activity of electron transport chain Complex I and a trend toward decreased Complex IV activity (Figure 3B,C). In aggregate, these biochemical results suggest a reduction in oxidative metabolism and a metabolic switch to aerobic glycolysis, or Warburg metabolism, in the MCT PAH model. These metabolic changes would be expected to contribute to observed reduction in RV contractile function. These metabolic findings are consistent with previous reports of decreased PDH activity and increased reliance on glycolysis in RV in PAH [6] Based on the therapeutic benefits of reactivating PDH using the PDK inhibitor dichloroacetate, this pathway accounts for much of the impairment of mitochondrial function in RVF-PAH [24]. Similarly, reduction of Complex I and IV activities have been reported in PAH lungs, where it is associated with a Warburg metabolic switch to uncoupled glycolysis [34,35]. However the literature on electron transport chain (ETC) function in RVF-PAH is less clear [36]. In contrast to our findings, Redout et al. showed that both expression and activity of Complex II were increased in RVF-PAH MCT rats and reported no modulation of protein expression of Complexes I, III, IV and V [36]. Conversely, in the RV of a porcine model of persistent pulmonary hypertension of newborn (PPHN), Saini-Chohan et al. showed a reduction of activity of mitochondrial complexes II and III and reduced protein levels of complexes II, III, and IV [23]. The disparities amongst studies could result from differences in the species, developmental stage and/or experimental model of PH studied. In addition, in most studies, there was a failure to subcategorize animals as having compensated versus decompensated RV. Impaired ETC activity is an additional intriguing potential contributor to RV dysfunction in PAH. Further investigation is necessary to determine if this results from dysfunction/damage to the proteins in the megacomplexes, substrate limitation, reduced mitochondrial copy number or some combination of these factors. While earlier implication of mitochondrial metabolic mechanisms in RVF were largely hypothesis driven, the current RNA sequencing data confirm a broad mitochondrial-metabolic dysregulation. Indeed, this is the most dysregulated pathway in the RV in PAH.

While the purpose of this paper was to provide an initial fingerprint of the RV transcriptome and identify dysregulated pathways, there are several robustly regulated genes in MCT RVF, which we have validated at the mRNA level (Table 3 and Table 4). While we did not explore their function in this initial study, their putative functions suggest possible roles in disease progression and consequently they may merit examination as therapeutic targets and/or biomarkers on RVF. For example, relevant to RV fibrosis, the most up-regulated gene in the RV of MCT rats encodes Periostin (*POSTN*; 4.5-fold). Periostin is a matricellular protein expressed, which is almost exclusively expressed in fibroblasts and which can regulate extracellular matrix interactions (ECM) [37] through the binding of various proteins. Periostin interacts directly with other ECM proteins, including many that are robustly up-regulated in the MCT RV dataset [collagen I (*COL1A1*; 1.9-fold), collagen V (*COL5A2*; 1.5-fold), Fibronectin (*FN1*; 2.4-fold) and tenascin-C (*TNC*; 2.5-fold)] [38,39,40]. Elevations of Periostin have already been identified in other cardiac diseases, including myocardial hypertrophy and ventricular remodelling where a fibrotic mechanism was identified [41]. Periostin has a role in the promotion of fibronectin secretion, and the fibronectin gene is also dysregulated in our dataset [39].

We placed the regulated genes in our list into enriched functional groups. Statistically-enriched terms describing functions related to “Mitochondria/Metabolism”, “Fibrosis”, and “Inflammation” emerged. In addition, we forced a GO:term for “Angiogenesis” into our final analysis, as this pathway was suggested by the literature to be relevant to RVF. We then surveyed independent RV microarray data from other laboratories. These studies had examined the transcriptomics of a mouse model of RVH, induced by pulmonary artery banding, and the RV of *BMPR2*-PAH patients. Because both prior studies relied upon few replicates (*n* = 2), we filtered this data with a lenient, uncorrected, threshold (*p*-value < 0.1). We concede that targets from this data have an unknown false-discovery rate, but note the extraordinary similarities, namely direction and relative magnitude of change, in our 4 functional dysregulated gene expression pathways (Figure 4A–D).

We further organized dysregulated genes so that those annotated in more than one of our enriched functions were identified. The gene, *ANNEXIN A1* (*ANXA1*; 1.80-fold), emerged as being both dysregulated and relevant to mitochondrial/metabolic, fibrotic, inflammatory and angiogenic pathways. *ANXA1* was downregulated −1.8-fold in RVF RVs. Anxa1 is a Ca^2+^-dependent, phospholipid-binding protein, which is primarily expressed in leukocytes. Glucocorticoids stimulate *Anxa1* expression, which inhibits phospholipase A2, blocks eicosanoid production, various leukocyte inflammatory events (epithelial adhesion, emigration, chemotaxis, phagocytosis, respiratory burst) and thereby mediates an anti-inflammatory effect [42]. Conversely, decreased *Anxa1* function can promote an endothelin-1 (*ET*-*1*)-induced inflammatory phenotype and PASMC proliferation in PH [43]. *Anxa1* overexpression inhibits *ET*-*1*-induced inflammatory cytokine secretion and PASMC proliferation (IL-6; IL-1β; TNFα); however, *Anxa1* is stimulated by IL-6, suggesting feedback between IL-6 and Anxa1 [44]. Upregulation of *Anxa1* in vivo, improves atherosclerosis (decreased lesion size, inflammation, improved plaque stability) [45,46,47], myocardial infarction (decreased infarct size, inflammation and increased survival) [48,49,50,51] and stroke (decreased infarct size, inflammation) [52,53,54,55]. In addition to increased inflammation, *Anxa1*-knockout mice exhibited significant increases of fibrosis eight weeks post-myocardial infarction [56]. Anxa1 has also been detected in the mitochondria and is associated with apoptosis [57,58]. In vitro experiments show that mitochondrial Anxa1 increases upon Ca^2+^ overload, contributing to physical interaction between the plasma membrane and mitochondria, thereby leading to apoptosis [57]. In tumor metabolism, oxidative and reductive glutamine metabolism was found to be significantly impaired in HIF-1α/Anxa1-deficient cells, and associated with lower proliferation [59]. This paradox could reflect regulation of Anxa1 function dependent of its localization (mitochondrial vs. cytoplasmic). Anxa1 deficiency has also been linked to impaired angiogenesis. *Anxa1*-KO mice exhibit a defect in angiogenesis and strongly impaired tumor growth. Aortic ring assays reveal that aortas from *Anxa1*^−/−^ mice exhibit impaired endothelial cell sprouting, which can be rescued by adenoviral expression of *Anxa1* [60]. Taken together, these observations confirm that Annexin A1 is involved in inflammation, fibrosis, “mitochondria/metabolism” and angiogenesis reported by our transcriptomic analysis. The fact that we have identified *Anxa1* downregulation in an unbiased manner in the MCT RV implies that this gene and its product may act as a lynchpin in MCT-induced RV failure.

Numerous genes relevant to mitochondrial function and biogenesis were downregulated in RVF. Expression of *Tfam*, a mitochondrial transcription factor that binds to mitochondrial DNA and facilitates transcription of the mitochondrial genome, is down-regulated 0.45-fold, in the MCT RV, consistent with impaired mitochondrial biogenesis. *Tfam* downregulation has been observed in human RVF [61]. The top down-regulated gene in the Mitochondria-Metabolic pathways is Cytochrome P450 2E1 (*Cyp2e1*; −3.8-fold expression). Cyp2e1 is a monooxygenase that is involved in metabolism of various endogenous and exogenous compounds. Cyp2e1 inhibition increases oxidative stress and apoptosis of cardiomyocyte in a murine dilated cardiomyopathy model and Cyp2e1 inhibition reduces cardiomyocyte apoptosis [62]. Thus, the observed decrease of *Cyp2e1* in MCT-RV might be a cardioprotective mechanism.

Dehydrogenase/reductase 7C (*Dhrs7c*) was also reduced (−2.8 fold). *Dhrs7c* has been demonstrated to be downregulated by adrenergic stimulation with both phenylephrine (α-adrenergic) and isoproterenol (β-adrenergic), and in several heart failure models, including biopsies from patients with heart failure [63].

Polypeptide *N*-acetyl-galactose-aminyl-transferase 13 (*Galnt13*) was also downregulated (−2.7-fold). *Galnt* is also downregulated in the blood of sickle-cell disease patients with pulmonary hypertension and could distinguish patients with and without increased RVSP with 100% accuracy [64]. Moreover, genetic association comparing patients with normal versus elevated tricuspid regurgitation jet velocity and pulmonary hypertension revealed 5 single nucleotide polymorphisms within this gene [64].

Thrombospondin4 (*Thbs4*; 4.4) is the highest regulated gene in the Fibroblast (and also in the Angiogenesis) functional group, and also one of the highest regulated genes overall; see independent validation in Table 3. Thbs4 is part of a family of glycoprotein that mediates cell-matrix interactions and is regulated in hypertrophic and failing hearts in various models and humans. Indeed, a single nucleotide polymorphism in the *Thbs4* gene has been associated with familial premature myocardial infarction [65]. While it is not clear whether *Thbs4* is regulated as part of a pathogenic or a protective response, it has been suggested as a mediator in fibrosis regulation and adaptive remodeling in the heart in response to pressure overload [66].

The second highest regulated gene in the Fibroblast functional list is latent-transforming growth factor beta-binding protein 2 (*Ltbp2*; 4.16). As their name suggests, *Ltbps* are critical for transforming growth factors (Tgf) to function [67]. Importantly, *Tgfb1* and *Tgfb2* are also upregulated in the MCT rat RV (1.2-fold and. 2.68 fold, respectively). *Ltbp2* is also robustly regulated in the failing RV in the SuHx model of PAH [68] and in humans this gene has been identified as upregulated in myocardial samples from heart failure patients [69].

While we have compared our data to transcript data from both mouse and human RVF, we acknowledge that this is not exhaustive and may describe specific rather than generic RV pathology and only at the transcript level. For example, comparison of our data with a recent proteomic analysis of RV from children with tetralogy of Fallot (TOF) [70] revealed only a small overlap between our data and theirs (17 proteins identified that have corresponding transcripts regulated in the rat MCT RV). While this paper also highlighted the significant enrichment of proteins involved in calcium signaling, our own functional analysis does not reveal this function as enriched. This poor concordance may reflect inherent differences between the proteome and transcriptome in these studies, or inherent biological differences between the infant human TOF RV and the “adult” rat MCT RV, or a combination of these factors, highlighting the importance of well-controlled comparative molecular analysis in order to identify pathology specific footprints.

In conclusion, transcriptomics reveals the molecular finger print of RVF in a well-phenotyped model of RV decompensation. This fingerprint is heavily characterized by mitochondrial dysfunction, and it appears to be pro-fibrotic, pro inflammatory, and anti-angiogenic. Our results confirm the previous observation made in the field [6]. Many of these dysregulated genes may be therapeutic targets or biomarkers and the changes in MCT-PAH are similar to those observed in a small sample of human PAH RVs, facilitating translation of preclinic studies. Proteomic and metabolic correlates of the disordered pathways identified by transcriptomics will be essential to evaluate which gene expression abnormalities are most relevant to disease pathogenesis or are most relevant to therapeutic targeting

## 4. Materials and Methods

Full Materials and Methods are available in the Supplementary Materials.

### 4.1. Monocrotaline-Induced PAH Animal Model

Experiments were conducted in accordance with the Canadian Council on Animal Care and approved by the Queen’s University Animal Care Committee (017-1714; 15 February 2018). Male Sprague-Dawley rats (~270 g, Charles River, Quebec, QC, Canada) received a single subcutaneous injection of monocrotaline (MCT; 60 mg/kg; C2401; Sigma-Aldrich, Oakville, ON, Canada), as previously described [27]. For additional detail, see Supplementary Materials.

### 4.2. Hemodynamic Parameters

Echocardiography: Non-invasive doppler, 2-dimensional, M-mode, tissue imaging and pulsed wave echocardiography were performed on anesthetized animals (5% isoflurane induction and maintained with 2% during procedures) using a high-frequency ultrasound system (Vevo 2100; Visual Sonics, Toronto, ON, Canada), as previously described [19] (see Supplementary Materials).

### 4.3. Right Heart Catheterization (RHC)

Invasive closed-chest RHC was performed to obtain RV pressure-volume (PV) loops. Briefly, animals were anesthetized with 5% isoflurane induction and maintained with 3% during procedures. During catheterization, rats were intubated and ventilated. A high-fidelity catheter (Scisence pressure-volume catheter; Transonic, London, ON, Canada) was advanced into the right ventricle (RV) via the jugular vein and right atria, in closed-chest rats. RV pressure and volume were recorded continuously using Scisense ADV500 Pressure-Volume Measurement System (Transonic) and LabScribe2 software (iWorx, Dover, NH, USA). For additional details, see Supplementary Materials.

### 4.4. Immunoblotting and Histology

See Supplementary Materials.

### 4.5. Mitochondrial Enzyme Activity

The activity of Pyruvate dehydrogenase (PDH), Complex IV and I were measured using enzyme activity dipstick assays from abcam (Cambridge, UK) according to the manufacturer’s recommendations (ab 109878; ab109720; ab109882) using 30–75 μg of protein extract, see Supplementary Materials.

### 4.6. RNA Sequencing

Right ventricles were dissected free from the LV and vasculature and ground with a mortar and pestle in liquid nitrogen. total RNA was extracted in TrIzol using Zymo DirectZol columns (Zymotech Inc., Austin, TX, USA). Libraries were generated using an Illumina RiboGold ribodepletion and Truseq stranded LT library generation kit (Illumina Inc., San Diego, CA, USA), and sequenced using the Illumina NextSeq550 Sequencer (Illumina). For additional details, see Supplementary Materials.

### 4.7. Statistical Analysis

All of the data are reported as mean ± SEM. Differences between groups were calculated using a two-tailed, Student’s *t*-test, corrected for multiple comparisons (Bonferroni). For additional details, see Supplementary Materials.

## Figures and Tables

**Table 1:** Hemodynamic characterization of RHF in MCT in MCT (*n* = 8) vs. Ctrl (*n* = 15) rats. CO, cardiac output; dP/dt_max_, peak rate pressure rise; dP/dt_min_, peak rate pressure decline; Ea, arterial elastance; EDV, end diastolic volume; EF, ejection fraction; Emax, maximal elastance; Emax/Ea, ventricular arterial coupling; HR, heart rate; mPAP, mean pulmonary arterial pressure; PAAT, pulmonary artery acceleration time; PRSW, preload recruited stroke work (slope of stroke work-EDV relationship); PVA/EDV, pressure volume area-EDV relathionship; RVSP, right ventricular systolic pressure; RVWF, right ventricle free wall; SW, stroke work; TAPSE, tricuspid annular plane systolic excursion; Tau Mirnsky, relaxation time constante calculated by Mirnsky method (time time requiered to RV pressure to fall to one half of its value at ESP); TPR, total pulmonary resistance (mPAP/CO). Unpaired *t*-test; data expressed as mean ± SEM. * *p* < 0.05.

	Ctrl (*n* = 15)	MCT (*n* = 8)
**Parameters**		
HR (BPM)	308.8 (7.33)	248.9 (18.85)
RVSP (mmHg)	23.51 (0.9742)	51.33 * (6.589)
mPAP (mmHg)	16.34 (0.5943)	33.31 * (4.019)
EDV (µL)	241.4 (21.12)	424.8 * (43.04)
SV (µL)	296.3 (8.3)	278.3 (23.97)
CO (µL/min)	91,650 (2672)	70,209 * (4425)
Ea (mmHg/µL)	0.1871 (0.01736)	0.26 * (0.01962)
Emax (mmHg/µL)	0.2828 (0.04747)	0.199 (0.05393)
PAAT (ms)	32.72 (0.8737)	21.17 * (0.7032)
RVFW (%)	106.7 (3.926)	28.33 * (8.476)
TPR (mmHg/mL/min)	0.1911 (0.007972)	0.4552 * (0.04644)
Fulton index	0.2725 (0.0054)	0.6354 * (0.0302)
**Systolic Indices**		
TAPSE (mm)	3.088 (0.05361)	2.16 * (0.1136)
EF (%)	66.36 (1.769)	52.69 * (2.343)
dP/dt_max_ (mmHg/s)	651.1 (22.4)	1048 * (117.8)
SW (mJoule)	0.4639 (0.03557)	0.9711 * (0.188)
Diastolic indices		
dP/dt_min_ (−mmHg/s)	577.4 (20.42)	1063 * (151.9)
Tau Mirnsky (mS)	29.11 (0.3685)	38.46 * (2.082)
Contractibility indices		
PRSW (mmHg)	14.12 (0.6412)	23.38 * (2.75)
PVA/EDV (mmHg)	0.001571 (0.0001373)	0.002625 * (0.0003239)
**Ventricular-Arterial Coupling**		
Emax/Ea	1.516 (0.1886)	0.7298 * (0.1884)

**Table 2:** Enriched functional analysis. List of significantly enriched terms that describe genes differentially expressed in rat RVF-PAH. List represents enriched terms that can broadly be classified as “Mitochondria/Metabolism”, “Fibrosis”, or “Inflammation”.

Term	Category	Function	Gene Count	*p* Value
GO:0005739~mitochondrion	GOTERM_CC_DIRECT	MitoMetabolism	414	1.85 × 10^−56^
Mitochondrion	UP_KEYWORDS	MitoMetabolism	196	4.16 × 10^−37^
GO:0005743~mitochondrial inner membrane	GOTERM_CC_DIRECT	MitoMetabolism	104	1.37 × 10^−22^
Mitochondrion inner membrane	UP_KEYWORDS	MitoMetabolism	65	7.55 × 10^−18^
transit peptide:Mitochondrion	UP_SEQ_FEATURE	MitoMetabolism	100	2.10 × 10^−17^
rno01100:Metabolic pathways	KEGG_PATHWAY	MitoMetabolism	279	3.38 × 10^−15^
rno00190:Oxidative phosphorylation	KEGG_PATHWAY	MitoMetabolism	56	5.89 × 10^−13^
GO:0005747~mitochondrial respiratory chain complex I	GOTERM_CC_DIRECT	MitoMetabolism	29	4.58 × 10^−12^
Oxidoreductase	UP_KEYWORDS	MitoMetabolism	109	3.00 × 10^−11^
Electron transport	UP_KEYWORDS	MitoMetabolism	31	7.73 × 10^−11^
GO:0055114~oxidation-reduction process	GOTERM_BP_DIRECT	MitoMetabolism	129	1.09 × 10^−8^
GO:0006979~response to oxidative stress	GOTERM_BP_DIRECT	MitoMetabolism	44	1.40 × 10^−8^
Ubiquinone	UP_KEYWORDS	MitoMetabolism	18	2.05 × 10^−8^
Respiratory chain	UP_KEYWORDS	MitoMetabolism	19	4.37 × 10^−7^
GO:0008137~NADH dehydrogenase (ubiquinone) activity	GOTERM_MF_DIRECT	MitoMetabolism	18	4.64 × 10^−7^
GO:0005759~mitochondrial matrix	GOTERM_CC_DIRECT	MitoMetabolism	43	6.38 × 10^−7^
Tricarboxylic acid cycle	UP_KEYWORDS	MitoMetabolism	12	9.93 × 10^−6^
GO:0000086~G2/M transition of mitotic cell cycle	GOTERM_BP_DIRECT	MitoMetabolism	16	1.11 × 10^−5^
GO:0006099~tricarboxylic acid cycle	GOTERM_BP_DIRECT	MitoMetabolism	14	1.16 × 10^−5^
GO:0006635~fatty acid beta-oxidation	GOTERM_BP_DIRECT	MitoMetabolism	18	1.27 × 10^−5^
GO:0050660~flavin adenine dinucleotide binding	GOTERM_MF_DIRECT	MitoMetabolism	22	5.42 × 10^−5^
FAD	UP_KEYWORDS	MitoMetabolism	26	5.49 × 10^−5^
GO:0031966~mitochondrial membrane	GOTERM_CC_DIRECT	MitoMetabolism	27	6.32 × 10^−5^
NAD	UP_KEYWORDS	MitoMetabolism	34	1.43 × 10^−4^
Flavoprotein	UP_KEYWORDS	MitoMetabolism	26	1.89 × 10^−4^
rno00020:Citrate cycle (TCA cycle)	KEGG_PATHWAY	MitoMetabolism	14	2.28 × 10^−4^
ATP synthesis	UP_KEYWORDS	MitoMetabolism	8	2.47 × 10^−4^
Mitochondrion outer membrane	UP_KEYWORDS	MitoMetabolism	20	8.23 × 10^−4^
TOTAL GENES (excluding duplicates)			647	
GO:0031012~extracellular matrix	GOTERM_CC_DIRECT	Fibrosis	72	5.83 × 10^−12^
GO:0030027~lamellipodium	GOTERM_CC_DIRECT	Fibrosis	47	3.85 × 10^−9^
GO:0005925~focal adhesion	GOTERM_CC_DIRECT	Fibrosis	90	4.02 × 10^−9^
Actin-binding	UP_KEYWORDS	Fibrosis	48	4.60 × 10^−9^
Cytoskeleton	UP_KEYWORDS	Fibrosis	109	8.70 × 10^−9^
GO:0051015~actin filament binding	GOTERM_MF_DIRECT	Fibrosis	39	5.91 × 10^−7^
Extracellular matrix	UP_KEYWORDS	Fibrosis	35	5.50 × 10^−6^
GO:0005604~basement membrane	GOTERM_CC_DIRECT	Fibrosis	28	9.53 × 10^−6^
GO:0005884~actin filament	GOTERM_CC_DIRECT	Fibrosis	24	9.77 × 10^−6^
GO:0042060~wound healing	GOTERM_BP_DIRECT	Fibrosis	34	1.09 × 10^−5^
rno04510:Focal adhesion	KEGG_PATHWAY	Fibrosis	54	1.34 × 10^−5^
GO:0031100~organ regeneration	GOTERM_BP_DIRECT	Fibrosis	27	1.77 × 10^−5^
Microtubule	UP_KEYWORDS	Fibrosis	39	2.53 × 10^−5^
GO:0005578~proteinaceous extracellular matrix	GOTERM_CC_DIRECT	Fibrosis	54	4.28 × 10^−5^
GO:0005874~microtubule	GOTERM_CC_DIRECT	Fibrosis	51	5.13 × 10^−5^
GO:0002102~podosome	GOTERM_CC_DIRECT	Fibrosis	13	1.49 × 10^−4^
rno04512:ECM-receptor interaction	KEGG_PATHWAY	Fibrosis	27	1.83 × 10^−4^
GO:0031252~cell leading edge	GOTERM_CC_DIRECT	Fibrosis	18	4.54 × 10^−4^
rno04810:Regulation of actin cytoskeleton	KEGG_PATHWAY	Fibrosis	50	5.84 × 10^−4^
Collagen	UP_KEYWORDS	Fibrosis	18	7.28 × 10^−4^
TOTAL GENES (excluding duplicates)			412	
Inflammatory response	UP_KEYWORDS	Inflammation	24	2.35 × 10^−5^
TOTAL GENES (excluding duplicates)			24	

**Table 3:** RNA sequencing validation. PCR validation in independent animals for the the top six genes related genes differentially expressed in RV of rat RVF-PAH vs. control (*n* ≥ 6 per group).

Gene	Functional Pathway	Sequencing (Ctrl/MCT)	PCR Validation (Ctrl/MCT)		
		(log) Mean Diff.	(log) Mean Diff.	SE of Diff.	Adjusted *p* Value
*POSTN*	Cell Adhesion	4.564263838	4.326	1.213	0.0065
*THBS4*	Angiogenesis Fibrosis	4.401212936	6.798	1.248	<0.0001
*LTBP2*	Fibrosis	4.16038822	3.545	1.112	0.0163
*RND1*	Cell Adhesion	2.639368653	2.687	0.9778	0.0451
*SCN3B*	Ion Transport	2.587455526	3.327	1.06	0.0183
*CNTFR*	Signal Transduction	−2.883345535	−5.146	1.352	0.0036

**Table 4:** RNA sequencing validation. PCR validation in independent animals for the top mitochondria-related genes differentially expressed in RV of rat RVF-PAH vs. control (*n* ≥ 6 per group).

Gene	Functional Pathway	Sequencing (Ctrl/MCT)	PCR Validation (Ctrl/MCT)		
		(log) Mean Diff.	(log) Mean Diff.	SE of Diff.	Adjusted *p* Value
*PRSS35*	mito/metabolism	4.23	4.31	0.8278	0.0001
*CDK1*	fibrosis mito/metabolism	2.11	0.5525	0.1917	0.0342
*ADAM12*	angiogenesis mito/metabolism	2.03	0.8509	0.2286	0.005
*BCAT1*	mito/metabolism	1.68	0.451	0.1891	0.1005
*LDHD*	mito/metabolism	−1.51	−0.8941	0.26	0.0089
*ADH1*	fibrosis mito/metabolism	−1.55	−0.8194	0.2765	0.0294
*EHHADH*	mito/metabolism	−1.56	−0.9721	0.2564	0.0038
*GSTA4*	mito/metabolism	−1.57	−0.7991	0.2441	0.0133
*ECI1*	mito/metabolism	−1.62	−0.8955	0.25	0.0063
*ACAA2*	mito/metabolism	−1.63	−0.8883	0.2471	0.0061
*MACROD1*	mito/metabolism	−1.8	−0.9501	0.2723	0.0079
*UCP3*	mito/metabolism	−1.86	−0.9768	0.3092	0.0057
*HADH*	mito/metabolism	−2.12	−0.9813	0.2654	0.0048
*CYP2E1*	mito/metabolism	−3.9	−6.394	1.469	0.0009
*DECR1*	mito/metabolism	−1.42	−0.8718	0.2272	0.0034

## Supplementary Materials

Supplementary materials can be found at http://www.mdpi.com/1422-0067/19/9/2730/s1.
