# Supplementary material for: Transcriptomic Signature of Right Ventricular Failure in Experimental Pulmonary Arterial Hypertension: Deep Sequencing Demonstrates Mitochondrial, Fibrotic, Inflammatory and Angiogenic Abnormalities
Source: Int J Mol Sci. 2018 Sep 12;19(9):2730. doi: 10.3390/ijms19092730 (PMC6164263; doi:10.3390/ijms19092730)
Supplement: Supplementary file 1 [file ijms-19-02730-s001.zip › ijms-349612-supplementary-for final/ijms-349612-Supplementary Materials.docx]

Supplementary Materials

Materials and Methods

Monocrotaline-induced PAH animal model

Experiments were conducted in accordance the Canadian Council on Animal Care and approved by the Queen’s University Animal Care Committee. Male Sprague-Dawley rats (~ 270 g, Charles River, QC, Canada) received a single subcutaneous injection of monocrotaline (MCT; 60 mg/kg; C2401; Sigma-Aldrich, Oakville, ON, Canada; n=13) or PBS (Thermo Fisher Scientific, Waltham, MA, USA; SH3025601; n=13). 4 weeks post-injection, animals were scarified, and tissues harvested. Experiments were designed and performed with attention to guidelines designed to enhance the robustness of preclinical investigation (see [73]). RNA-Seq has been performed on 5 control rats and 6 MCT rats. Complex I/IV and PDH activities as well as Western blots, have been performed in 5 control rats and 4 MCT rats (animals used for RNA seq). PCR validation has been performed in a separate additional cohort of 7 control rats and 7 MCT rats. Hemodynamic parameters were performed on 15 control rats and 8 MCT rats which include all the animals used for RNA-Seq. In total this study evaluated 15 control and 13 monocrotaline rats.

Hemodynamic parameters

Echocardiography: Non-invasive doppler, 2-dimensional, M-mode, Tissue imaging and pulsed wave echocardiography were performed on anesthetized animals (5% isoflurane induction and maintained with 2% during procedures) using a high-frequency ultrasound system (Vevo 2100; Visual Sonics, Toronto, ON, Canada), as previously described[19]. The following variables were measured: pulmonary artery acceleration time (PAAT), tricuspid and mitral annular plane systolic excursion (TAPSE; MAPSE), S-wave, e’-wave, E-wave. RVFW systolic thickening was calculated as (RVFW_systole_ -RVFW_diastole_)/RVFW_diastole_. Diastolic function of the left ventricle was assessed by E/e’ ratio.

Right heart catheterization (RHC)

Invasive closed-chest RHC was performed to obtain RV pressure-volume (PV) loops. Briefly, animals were anesthetized with 5% isoflurane induction and maintained with 3% during procedures. During catheterization, rats were intubated and ventilated. A high-fidelity catheter (Scisence pressure-volume catheter; Transonic, London, ON, Canada) was advanced into the right ventricle (RV) via the jugular vein and right atria, in closed-chest rats. RV pressure and volume were recorded continuously using Scisense ADV500 Pressure-Volume Measurement System (Transonic, London, ON, Canada) and LabScribe2 software (iWorx, Dover, NH, USA). Pressure volume loops (PV-loop) were generated at baseline and during unloading by abdominal aorta section. RV systolic pressure and end-diastolic pressure (RVSP and RVEDP, respectively) were directly obtained from the pressure trace. Stroke volume (SV), end diastolic volume (EDV), end systolic pressure (ESP) was obtained from the PV-loop. Total pulmonary resistance (TPR) was then calculated as mPAP/CO, where CO is cardiac output calculated as (RV end-diastolic volume – RV end-systolic volume) $\times$ heart rate (HR), and mPAP is the mean pulmonary artery pressure estimated as 0.61 $\times$ RVSP + 2[74]. Diastolic function was assessed by peak rate pressure decline (dp/dtmin); Tau Mirnsky, relaxation time constant calculated by Mirnsky method which evaluates the time needed for RV pressure to fall to one-half of its value from peak rate of RV pressure fall (dP/dt_min_)[75]. Systolic function was measured by calculating by calculating the peak rate of pressure rise (dp/dtmax), stroke work (SW), and ejection fraction (EF). Preload recruitable stroke work (PRSW) and the pressure volume area (PVA)-EDV relationship (PVA/EDV) were used as contractility indices. Arterial elastance (Ea) was calculated as the ratio of ESP to SV and maximal elastance (Emax) was determined as the slope of the end systolic pressure volume relationship after altering preload. These parameters were used to calculate ventricular-arterial coupling (Emax/Ea). The Fulton index was used to quantify RVH (RV/LV+septal weight).

Immunoblotting

Proteins were extracted via cell lysis and 80 µg protein was loaded to SDS-PAGE gel for immunoblotting. Images were analyzed with ImageJ (National Institutes of Health, Bethesda, MD, USA). CD31 (ab64543) and CD68 (ab31630) antibodies where purchased from abcam (Cambridge, UK).

Histology

Hearts were fixed in 10% buffered formalin. Picrosirius red staining was performed to assess collagen deposition. Staining was performed on 5 μm paraffin-embedded heart sections. Images were acquired with a Leica DM4000B LED microscope with a 20x objective (Leica Microsystems; Wetzlar, Germany). Picrosirius Red was quantified as previously published[76].

RNA sequencing

Right ventricles were dissected free from the LV and vasculature and ground with a mortar and pestle in liquid nitrogen. total RNA was extracted in TrIzol using Zymo DirectZol columns (Zymotech Inc, Austin TX, USA). Libraries were generated using an Illumina RiboGold ribodepletion and Truseq stranded LT library generation kit (Illumina Inc., San Diego, CA, USA), and sequenced using the Illumina NextSeq550 Sequencer (Illumina). The resulting .fastq files will be deposited to the Gene Expression Omnibus: GEO Accession: GSE119754. Raw reads were pre-processed using Cutadapt (a Python Command Line Tool, Dortmund, Germany), aligned using HISAT2 and assembled using StringTie (<http://ccb.jhu.edu/software.shtml>). Raw count data was produced from the aligned reads using featureCounts (an R package, <http://bioinf.wehi.edu.au/featureCounts/>), and then differential expression analysis using edgeR (*Empirical Analysis of Digital Gene Expression Data in R*, <https://bioconductor.org/packages/release/bioc/html/edgeR.html>).

Statistical analysis

All of the data are reported as mean ± SEM. Differences between groups were calculated using a two-tailed, Student’s t-test, corrected for multiple comparisons (Bonferroni). Statistical analyses were performed using GraphPad Prism 7.0d (GraphPad Software, La Jolla, CA, USA). A p-value <0.05 was considered statistically significant. The methods for statistical filtering of RNAsequencing data are detailed in the Results section.

Supplementary Tables Legends

**Supplementary Table 1: Gene List.** List of 2,546 transcripts that were significantly and differentially regulated in the rat RV as a consequence of MCT treatment. Data is annotated to include Ensemble ID, Gene symbol, log Fold change, Absolute fold change and direction, log CPM (log counts per million, a proxy for expression), p-value and corrected p-value (FDR). This dataset also includes columns that indicate whether each gene is functionally classed as Angiogenesis, Fibroblast, Mitochondria/Metabolic or Inflammation. Data is also presented for the mouse RV and the human RV; this data is not filtered with respect to p-value at this stage.

**Supplementary Table 2: Functional Analysis.** Raw result from the Database for Annotation, Visualization and Integrated Discovery (DAVID)[30] analysis. This data is presented as category (for example, GOTERM, UP_KEYWORD, KEGG PATHWAY), the Term that describes the data (e.g. GO:0005739~mitochonrion), the count of how many genes are captured in each Term, the % of total genes in the list, Ensemble IDs of genes, Fold enrichment of term, and multiple test corrected p values using Benamini. .

**Supplementary Table 3: Filtered Functional Analysis.** Table of ‘Terms’ that we assigned as either Mitochondrial/Metabolic, Fibrosis or Inflammation.

**Supplementary Table 4: Angiogenesis - GO 0001525.** List of targets that belong to the functional group for Angiogenesis.

**Supplementary Table 5: Mouse Array.** Data obtained as the result of analysis performed on the mouse data (Accession: GSE30428).

**Supplementary Table 6: Human Array.** Data obtained as the result of analysis performed on the human data (Accession: GSE67492).

**Supplementary Table 7: Common Rat-Mouse RV.** Genes that are commonly expressed in the rat MCT RV and the mouse PAB RV (significance cut-off p<0.1 for mouse data).

**Supplementary Table 8: Common Rat-Human RV.** Genes that are commonly expressed in the rat MCT RV and the human BMPR2 RV (significance cut-off p<0.1 for human data).

**Supplementary Table 9: Common Rat-Mouse-Human RV.** Genes that are commonly expressed in the rat MCT RV, the mouse PAB RV and the human BMPR2 RV (significance cut-off p<0.1 for mouse and human data).

**Supplementary Table 10: Fibroblasts.** List of genes that are common to rat MCT RV, mouse PAB RV and human BMPR2 RV that are functionally defined as ‘Fibroblast’ (significance cut-off p<0.1 for mouse and human data).

**Supplementary Table 11: Mitochondria Metabolic.** List of genes that are common to rat MCT RV, mouse PAB RV and human BMPR2 RV that are functionally defined as ‘Mitochondria Metabolic’ (significance cut-off p<0.1 for mouse and human data).

**Supplementary Table 12: Inflammation.** List of genes that are common to rat MCT RV, mouse PAB RV and human BMPR2 RV that are functionally defined as ‘Inflammation’ (significance cut-off p<0.1 for mouse and human data).

**Supplementary Table 13: Angiogenesis.** List of genes that are common to rat MCT RV, mouse PAB RV and human BMPR2 RV that are functionally defined as ‘Angiogenesis’ (significance cut-off p<0.1 for mouse and human data).
